# Association of initial intracellular signalling pathway and cytokine level with early mortality in severe sepsis patients

**DOI:** 10.1186/cc14049

**Published:** 2014-12-03

**Authors:** KC Lie, D Widodo, S Lardo, Eppy, R Sinto

**Affiliations:** 1 Division of Tropical and Infectious Diseases, Department of Internal Medicine, Faculty of Medicine, Universitas Indonesia, Jakarta, Indonesia; 2 Department of Internal Medicine, Gatot Subroto Indonesia Central Army Hospital, Jakarta, Indonesia; 3 Department of Internal Medicine, Persahabatan Hospital, Jakarta, Indonesia

## Introduction

Sepsis occurs as a result of systemic inflammatory process. The release of bacterial components to the systemic circulation leads to activation of inhibitor kappa B kinase (IKK)-β and nuclear factor kappa B (NF-κB) through phosphorylation and ubiquitination. Excessive inflammatory process with maladaptive host's immune response leads to organ dysfunctions and death [1,2]. This study was designed to investigate association of the initial level of intracellular signalling pathway and cytokine involved in sepsis pathophysiology (that is, IKK-β, NF-κB, tumour necrosis factor (TNF)α) and 72-hour (early) mortality in severe sepsis patients.

## Methods

A prospective cohort study was conducted in severe sepsis patients (aged 18 years and older) admitted to the Emergency Unit of Cipto Mangunkusumo Hospital, Persahabatan Hospital, and Gatot Subroto Indonesia Central Army Hospital, Jakarta, Indonesia. All blood samples for intracellular signalling pathway (that is, IKK-β, total NF-κB, phospho NF-κB) and cytokine (that is, TNFα) were collected and measured using the ELISA method during first 24 hours of inclusion. Patients' outcome was observed during first 72 hours after inclusion. Mann-Whitney test was used to analyse the median difference of IKK-β, total NF-κB, phospho NF-κB level in two groups based on 72-hour survival. The *t *test was used to analyse the mean difference of TNFα levels in both groups.

## Results

Subjects consisted of 90 patients. Early mortality developed in 27 subjects. Baseline characteristics of survival and death subjects are shown in Table 1. The initial intracellular signalling pathway and cytokine parameters level are shown in Table 2. There was a significant higher median first 24 hours IKK-β and total NF-κB level in survival subjects compared with death subjects (*P *= 0.025 for median IKK-β and *P *= 0.036 for median total NF-κB). There was no significant difference of initial phospho NF-κB and TNFα level in survival and death subjects.

**Table 1:** Baseline characteristics of the subjects

Characteristic	Survival subjects (*n *= 63)	Death subjects (*n *= 27)
Mean age (years)	52.98 ± 15.38	50.19 ± 13.46
Sex		
Male	23 (36.5)	12 (44.4)
Female	40 (63.5)	15 (55.6)
Sepsis severity		
Severe sepsis	42 (66.7)	13 (48.1)
Septic shock	21 (33.3)	14 (51.9)
Mean APACHE II score	13.03 ± 5.27	14.37 ± 6.55
Comorbidity		
Without comorbidity	4 (6.3)	2 (7.4)
With comorbidity(s)	59 (93.7)	25 (92.6)
Source of infection		
Respiratory tract	45 (71.4)	24 (88.9)
Intraabdominal	4 (6.3)	1 (3.7)
Skin and soft tissue	5 (7.9)	1 (3.7)
Urinary tract	2 (3.1)	1 (3.7)
≥ 2 sources	7 (11.3)	0
Mean procalcitonin (pg/ml)	21.47 ± 44.30	30.26 ± 17.29
Mean baseline lactate (mmol/l)	3.10 ± 1.77	4.89 ± 2.57

Data presented as *n *(%) or mean ± standard deviation. APACHE, Acute physiology and chronic health evaluation.

**Table 2:** Initial intracellular signalling pathway and cytokine level based on 72-hour survival

Parameter	Survived subjects (*n *= 63)	Death subjects (*n *= 27)	*P *value
IKK-β^a^	0.10 (0.03)	0.12 (0,03)	0.025
Total NF-κB^a^	0.08 (0.08)	0.12 (0.08)	0.036
Phospho NF-κB^a^	0.10 (0.38)	0.33 (0.38)	0.579
TNFα^b^	9.10 (7.80)	10.91 (11.74)	0.393

^a^Median (interquartile range) in optical density, Mann-Whitney test. ^b^Mean (standard deviation) in pg/ml, *t *test.

## Conclusion

There is a significant lower initial IKK-β and total NF-κB level in severe sepsis patients surviving on 72-hour observation. There is a tendency of lower initial phospho NF-κB and TNFα level in severe sepsis patients surviving on 72-hour observation.
